# Clinical and Genetic Study on a Chinese Patient with Infantile Onset Epileptic Encephalopathy carrying a *PPP3CA* Null Variant: a case report

**DOI:** 10.1186/s12887-020-02213-7

**Published:** 2020-06-27

**Authors:** Sai Yang, Xiang Shen, Qingyun Kang, Xiaojun Kuang, Zeshu Ning, Shulei Liu, Hongmei Liao, Zhenhua Cao, Liming Yang

**Affiliations:** 1 Department of Pediatrics, Hunan Children’s Hospital, No.86 Ziyuan Road, Changsha, 410007 Hunan China; 2 Running Gene Inc., Haohai Mansion, No.7 Shangdi 5th Street, Haidian District, Beijing, China

**Keywords:** PPP3CA, IECEE1, seizure, Video-EEG, Case report

## Abstract

**Background:**

*PPP3CA* gene encodes the catalytic subunit A of a calcium-dependent protein phosphatase called calcineurin. However, two distinct mechanisms in PPP3CA deficiency would cause two clinically different diseases. Gain-of-function mutations in the autoinhibitory domain at the C-terminus would cause ACCIID that stands for arthrogryposis, cleft palate, craniosynostosis and impaired intellectual development. While loss-of-function mutations in *PPP3CA* would cause infantile or early childhood onset epileptic encephalopathy1, named as IECEE1. IECEE1 is a severe epileptic neurodevelopmental disorder and mainly characterized by psychomotor delay. Here, we report a Chinese patient who was clinically and genetically diagnosed as IECEE1. We also extensively analyzed electroencephalogram (EEG) features of the patient in this study.

**Case presentation:**

A 2-year-old Chinese patient who had recurrent polymorphic seizures was clinically and genetically diagnosed as IECEE1. A frameshift variant c.1283insC (p.T429NfsX22) was identified in this case. Multiple types of abnormal features were observed in the EEG, comparing with the previous reports.

**Conclusions:**

These findings could expand the spectrum of *PPP3CA* mutations and might also support the diagnosis and further study of IECEE1.

## Background

Infantile or early childhood onset epileptic encephalopathy1 (IECEE1, OMIM#617711) and arthrogryposis, cleft palate, craniosynostosis, impaired intellectual development (ACCIID, OMIM#618265) are two autosomal dominant diseases caused by mutations in *PPP3CA* gene. These two diseases are both related to neurodevelopmental disorders but differ in clinical manifestations. IECEE1 is characterized by global developmental delay or even regression with mental retardation and speech difficulty. Patients with IECEE1 present refractory seizures with varying onset age, and some of them also have hypotonia or visual disorders [1]. However, ACCIID is mainly characterized by short stature, intellectual disability, and malformations, including arthrogryposis, cleft palate, craniosynostosis, or micrognathia. Other bony abnormalities, such as tubular bones and perinatal fractures, have also been observed. Seizures were only reported in individual cases, and the frequency of attack is relatively lower than IECEE1 [2]. A research by Mizuguchi et al. has demonstrated that these two disorders are different in mechanism. Missense mutations in the autoinhibitory domain (AID) at C-terminus might enhance the function of PPP3CA, leading to ACCIID. While other missense mutations or null variants (including nonsense and frameshift mutations) might damage the function of PPP3CA, resulting in IECEE1 [2].

To date, 14 mutations from 6 papers have been reported [1–6], including 8 missenses, 2 nonsenses, 1 splicing mutation, and 2 small insertions associated with IECEE1. Here, we reported our experience in the diagnosis of a 2-year-old Chinese IECEE1 patient, using the clinical and genetic examinations. We described video-electroencephalogram (V-EEG) features of the patient, reviewed reported cases carrying *PPP3CA* mutations and compared the phenotypes with those of patients who harboured the same variant.

## Case presentation

### Clinical information

A 2-year-old boy was admitted to our department due to generalized tonic-clonic seizures. He is the first child but the third pregnancy for his non-consanguineous Chinese parents. His mother had two miscarriages before, and this pregnancy was unremarkable. The patient was born via cesarean section at 40-week-3-day gestation with a birthweight of 3.2 kg (50th percentile) and length of 50 cm (50th percentile). His head circumference was 30.9 cm (50th percentile at birth). Refractory eczema was first observed at 6 months. Following the treatment with Desonide cream, the eczema totally relieved after 18 months. The patient first got his head control at 3 months, sat independently at 7 months and walked at 20 months. When he was admitted to the hospital, the height was 95 cm (75th percentile), the weight was 14 kg (50th–75th percentile) and the head circumference was 49 cm (50th percentile). No facial abnormalities were observed. The patient could walk alone but presented a tip-toe gait. He could respond to his name and chase objects. Language delay was significant with meaningless sound and no word. No abnormality was identified from blood and urine routine test, chest CT scans, magnetic resonance imaging (MRI), abdominal ultrasound analysis or color doppler flow imaging ultrasonography (CDFI). No family history was reported.

At the age of 2, the patient experienced his first generalized tonic-clonicseizure lasting for 20 min. No triggering factors were identified. Since it was the first seizure, no specific treatment was given to the patient. However, a half month later, the myoclonic seizures recurred with a frequency of 50–60 times per day. During the episodes, he remained conscious with limb jerks, nodding head, and rolling eyes. No fever, cough, or diarrhea were observed. The patient also had no alteration in movement or personality. Since the intermittence between these two unprovoked seizures was more than 24 h, the antiepileptic drug (AED) valproate (VPA) was given (10 mg/kg/day, divided q12H). The dosage was then added up to 15 mg/kg/day (divided q12H) after 5 days. The patient was then discharged as the epilepsy relieved. The valproate was continued with a dosage of 20 mg/kg/day (divided q12H). One month and a half later, the dosage was added up to 30 mg/kg/day (divided q12H). However, 4 days after the dosage increased, the seizures recurred with the frequency of 10–30 times/day. The dosage of VPA was further increased to 40 mg/kg/day (divided q12H) after epileptic spasms. Atypical absence seizures were identified. The parents refused ACTH treatment, and they still presented epileptic spasms 10 times/day. One month later, levetiracetam (LEV) 20 mg/kg/day, divided q12H (which was further increased to 30 mg/kg/day) and ketogenic-diet combined with the previous VPA therapy was commenced [7]. The patient still presents nod 10–20 times/day till now.

### V-EEG characteristic

**Fig. 1:**
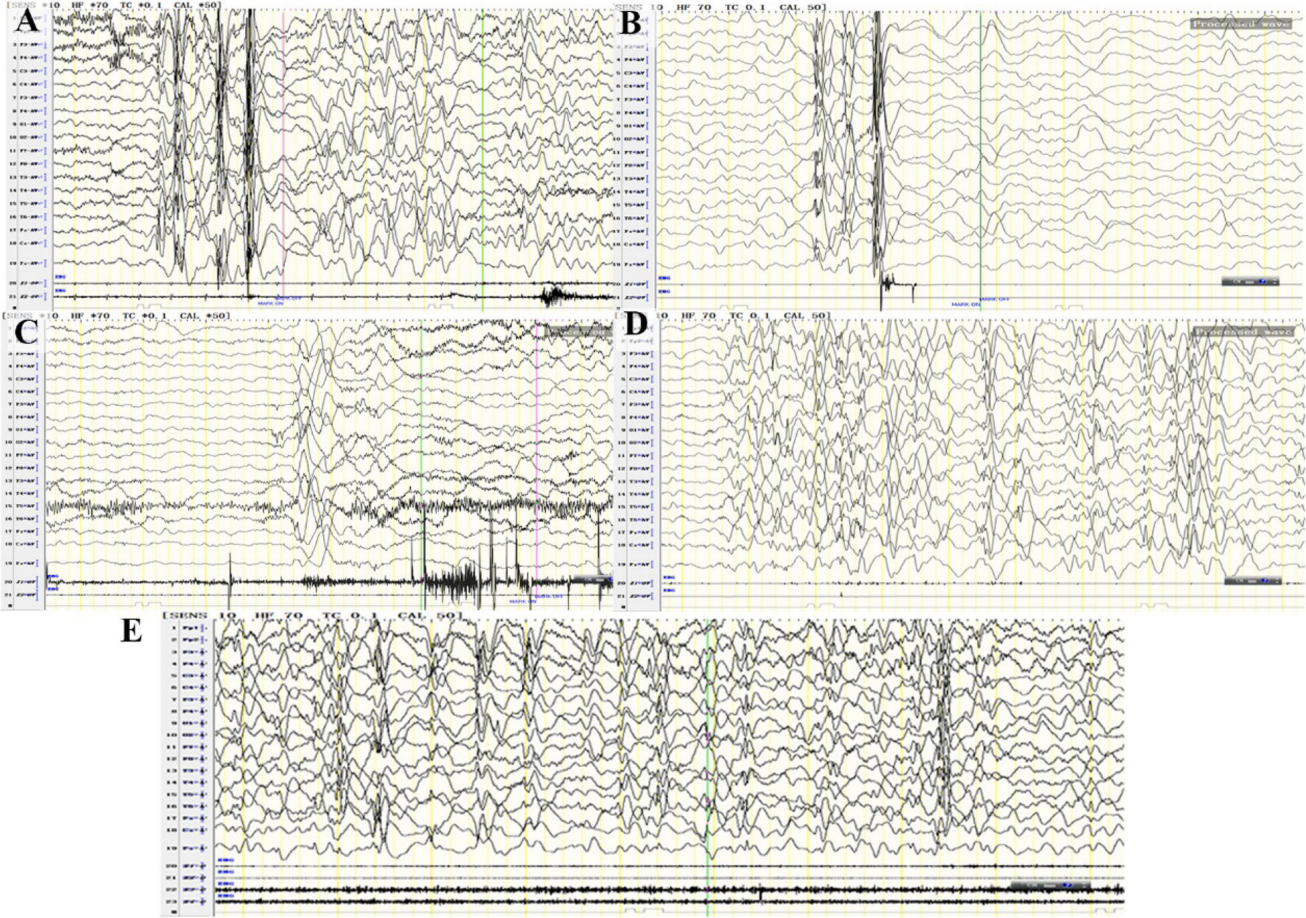
The tracking EEG result of the patient. **a** At 2 years 1 months, the EEG result of the patient during wakefulness showed generalized myoclonic seizures with generalized slow spike-and-wave with high amplitude. **b**-**d** The EEG result of the patient at 2.5 years old. Sleeping myoclonic seizures accompanied by bursts of generalized slow spike-and-wave (**b**) and waken epileptic spasms accompanied by generalized slow waves of high amplitude and fast waves of low amplitude (**c**) were detected. Fragmented hypsarrhythmic waves were observed during the interval between spasms (**d**). **e** Irregular slow spike-and-waves were presented during the atypical absence seizures of the waken patient detected in the last follow-up EEG

V-EEG was conducted and the data was reviewed and interpreted (Fig. 1). At the initial onset stage (2 years and 1 month old), the V-EEG monitoring showed slower background rhythms and multi-focal diffuse high amplitude δ activity (2–3 Hz) in stage wake (W). The type of seizure was generalized myoclonic seizure. The patient presented frequently single or double nods. The patient normally retained consciousness. Generalized bursts of slow spike-and-wave discharge with the highest amplitude could be seen when myoclonic seizures attacked.

Five months later, the follow-up V-EEG demonstrated slower background rhythms and frequent clusters of generalized or multi-focal poly-spike discharges. During the interictal stage, fragmented atypical hypsarrhythmia discharges appeared. The observed seizure of the patient was developed into three types this time: generalized myoclonic seizures, epileptic spasms and atypical absence seizures. **For generalized myoclonic seizures**, single or double rapid proximal jerks in one lateral upper limb could be observed during the episode. V-EEG results revealed synchronous bursts of generalized slow spike-and-waves accompanied by 50 ms bursts of myoelectricity at deltoid muscles. **For epileptic spasms**, the patient remained awareness and consciousness during the episodes. Nodding, bending or upward staring could be observed. Each episode lasts very short for only 1–1.5 s, and was isolated and discontinued. V-EEG result demonstrated complex of high amplitude slow waves and low-amplitude high-frequency activities during the episodes. Synchronous EMG demonstrated 0.5–1 s bursts of myoelectricity followed by 1–2 s transient generalized voltage suppression at rhomboid muscles. **For atypical absence seizures**, the patient would have less movement and slow mild head bobbing, lasting for 8–30 s per episode. V-EEG revealed rhythmic generalized slow spike-and-wave discharges activity (1.5–2.5 Hz).

A month later, the latest follow-up V-EEG demonstrated slower background rhythms and frequent clusters of generalized or multi-focal sharp wave, spike-and-wave, as well as slow spike-and-wave discharges. During the interictal stage, generalized rhythmic slow spike-and-wave with irregular spikes was presented. Generalized myoclonic seizure, epileptic spasms and atypical absence seizures can still be identified.

### Next-Generation Sequencing

Whole-exome sequencing (WES) and Sanger sequencing were performed by Running Gene Inc. (Beijing, China) using their standard process, which is available in the previous report [8]. The variant c.1283insC (GRCh37/hg19, NM_000944) of *PPP3CA* was identified in the proband. This variation would cause a frameshift p.T429NfsX22 in downstream sequence of the protein, leading to alterations in protein structure. Since this variation is a frameshift (PVS1), a de novo variation that carried by neither of the parents (PS2), a variation that is absent from controls (PM2) and a disease-causing variation that has been identified before [5], it was classified as ‘pathogenic’ according to the ACMG guidelines [9].

## Discussion and conclusion

IECEE is an autosomal dominant disorder characterized by refractory seizures, psychomotor development delay and intellectual disability, which is caused by mutations in *PPP3CA* gene. Protein encoded by *PPP3CA* gene mainly consists of four domains, including the core catalytic domain, the calcineurin B binding domain, the calmodulin-binding domain and the autoinhibitory domain (AID) [10, 11]. It acts as one isoform of the catalytic subunit of a phosphatase called calcineurin, an essential regulator of intracellular Ca^2+^-mediated signals, which has been reported to be related to synaptic vesicle endocytosis and transmission [12–15]. If the function of PPP3CA was destroyed, calcineurin would be inactivated, causing IECEE syndrome. Thus, the report of mutations in *PPP3CA* shows great significance in the clinical and genetic diagnosis of IECEE1. Here we report the clinical and genetic features of a male child who was genetically diagnosed as IECEE1.

### V-EEG characteristics of the IECEE1 patient

As V-EEG features of IECEE1 patients have not been systematically characterized, we traced V-EEG features of our patient in different stages and summarized the features. The patient had slower background rhythms in different stages of IECEE1. Sharp, spike, poly-spike, sharp-slow and spike-slow waves were observed in various regions. Fragmented hypsarrhythmia brain waves were also observed. Generalized tonic-clonic seizures, generalized myoclonic seizures, epileptic spasms and atypical absence seizures were all observed.

We also managed to identify the development procedure of the patient’s seizures by tracing his V-EEG. The patient first presented generalized tonic-clonic seizures, and later transferred to generalized myoclonic seizures. Increased dosage of VPA treatment failed to control the seizures of the patient, epileptic spasms and atypical absence seizures developed over time. The patient showed poor prognosis even treated with increased dosage of LEV and VPA. Finally, the patient displayed very complex epilepsies.

### Genetic analysis

**Fig. 2:**
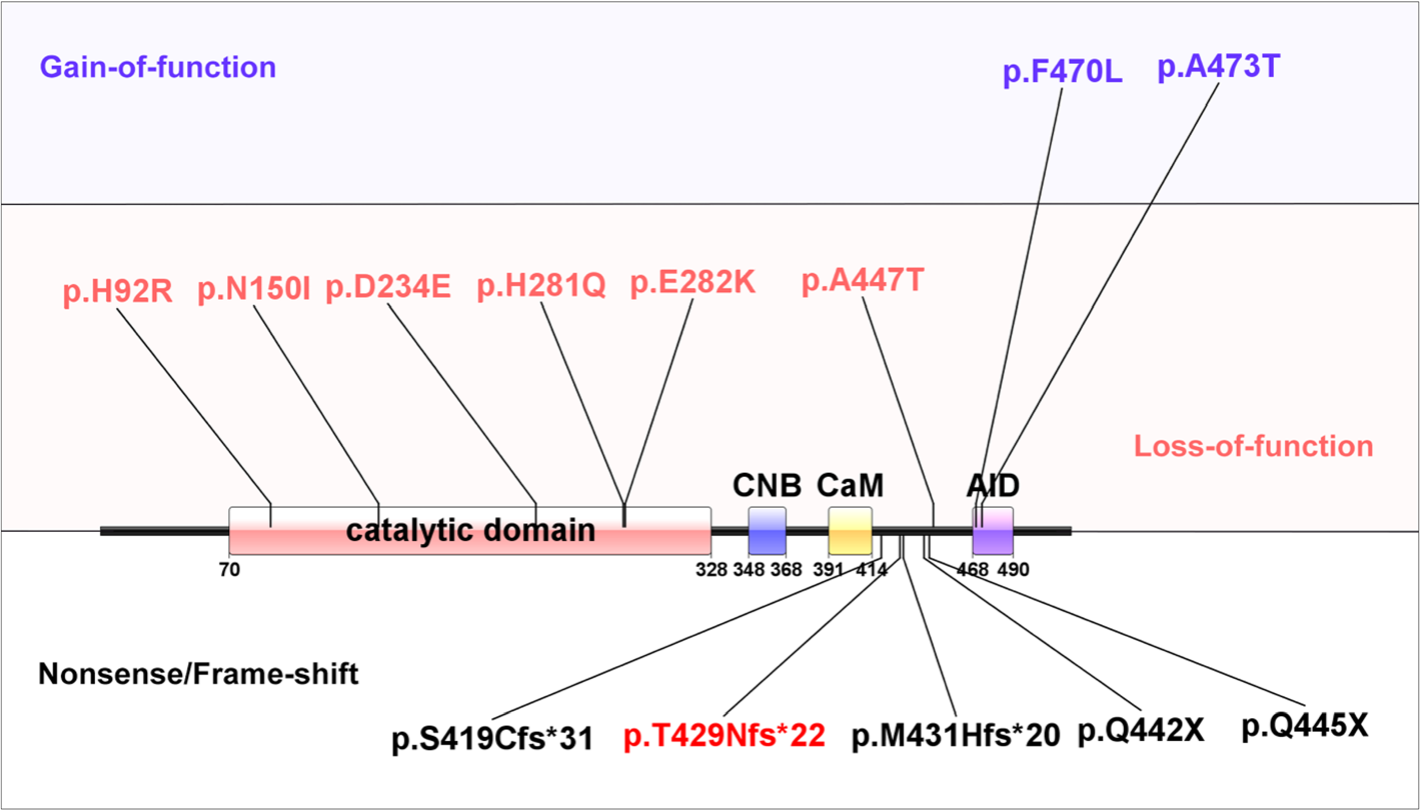
The mutation spectrum of PPP3CA. All recorded disease-causing mutations in HGMD database. The gain-of-function missense mutations, loss-of-function missense mutations and nonsense/frameshift mutations were marked separately. The frameshift mutation in our study was marked in red

According to previous reports, most of the documented mutations in *PPP3CA* are missense mutations (Fig. 2), resulting in the gain or loss of function [1–3, 6]. Among them, only missense located at the upstream of AID domain displayed a loss-of-function effect and presented IECEE1 phenotypes in patients. The patients carrying nonsense or frameshift variant (our case included) also presented similar phenotypes of IECEE1 [2, 3]. Thus, the variant found in our case, located before the disease-causing frameshift mutation, p.M431Hfs*20, is predicted to cause IECEE1.

Our case is the second case with variant p.T429NfsX22 of *PPP3CA*. Phenotypes of these two cases (Table 1) were compared. The two cases were identified to display similar clinical features. Both cases presented epileptic spasms with hypsarrhythmia in EEG, and MRI results were normal. However, some reported abnormal EEG displays, including abnormal background, generalized spike-and-slow waves, and poly-spikes, were only identified in our case. Furthermore, we clearly presented the development of the seizure of our case, which was not mentioned by Li et al.

**Table 1:** The clinical information of patients carrying pathogenic mutations in PPP3CA

Case	Our case	Li et al. 2019 Case 6	Cases summarized by Qian et al. 2018
**Age**	2.5y	3y 8 m	8.46y (±7.14)
**Gender**	M	F	4 M/10F
**Mutation**	c.1283insC	c.1283insC	multiple variants
**Amino acid alteration**	p.T429NfsX22	p.T429NfsX22	multiple variants
**Mutation type**	Frameshift	Frameshift	nonsense/frameshift/missense
**Inheritance**	De novo	De novo	De novo
**Onset age**	2y	1y1m	14.18 m (±15.46)
**EEG**	Slow background waves, PS, GSW, hypsarrhythmia, spasms	hypsarrhythmia, MFD and spasms	including MFD, PS, GSW, Hypsarrhythmia, Abnormal background and other abnormal waveforms
**MRI**	Normal	Normal	Some are normal. Some presented abnormalities including ventricular dilatation, white matter change and small hippocampal.
**Others**	Eczema, speech delay	Mild developmental delay, poor speech, and mild hypotonia	–

*MFD multifocal epileptiform discharges; *PS* polyspikes; *GSW* generalized spike and slow waves

In conclusion, we reported a Chinese IECEE1 patient with a frameshift variant p.T429NfsX22 in *PPP3CA*, expanding the variety of *PPP3CA* mutations. We also concluded the characteristics of disease-associated pathogenic mutations and shared the analysis of intensive EEG features, which may support the further diagnosis of IECEE1.

## Data Availability

If you need more data or materials about this case, please contact the corresponding author Dr. Liming Yang.

## Abbreviations

IECEE1: Infantile or early childhood onset epileptic encephalopathy1
ACCIID: Arthrogryposis, cleft palate, craniosynostosis and impaired intellectual development
V-EEG: Video-electroencephalogram
AID: Autoinhibitory domain
AED: Antiepileptic drug
VPA: Valproate
ACTH: Adrenocorticotropic hormone
MRI: Magnetic resonance imaging
CDFI: Color doppler flow imaging ultrasonography
WES: Whole-exome sequencing
NFATC1: Nuclear factor of activated T-cells, cytoplasmic 1
ELK1: ETS domain-containing protein Elk-1
NMD: Nonsense-mediated mRNA decay
LEV: Levetiracetam
